# Re-assessing viologens for modern bio-electrocatalysis†

**DOI:** 10.1039/d4sc02431a

**Published:** 2024-05-16

**Authors:** Desmond Ato Koomson, Jake H. Nicholson, Alex P. S. Brogan, Leigh Aldous

**Affiliations:** a Department of Chemistry, King's College London Britannia House London SE1 1DB UK alex.brogan@kcl.ac.uk leigh.aldous@kcl.ac.uk

## Abstract

Viologens, 1,1′-disubstituted-4,4′-bipyridinium salts, are organic redox species that can be used in place of NADPH as mediators for redox enzymes. In this study, using the reduction of oxidized glutathione by glutathione reductase as a model system, a rationally designed library of viologens covering a range of polarities and functional groups were explored as electron transfer mediators for bio-electrocatalysis. Through a series of electrochemical investigations, the reduction potential was found to be the primary determining factor for electron transfer between the viologen and enzyme. Through enhancing the solubility of viologen such that the fully reduced state remained soluble, we demonstrate a much-widened window of useable viologen potentials. In doing so, we describe for the first time a highly efficient electron transfer to a flavoenzyme promoting the catalytic reaction in the absence of co-factors. As such, our study provides a platform for broadening the scope for using viologens as mediating agents for electrochemically-driven enzymatic processes.

## Abstract 2

Through a rationally designed library of viologens, we show that catalytic efficiency of a model flavoenzyme is strongly correlated to the reduction potential. This work demonstrates viologens as versatile mediators for biocatalytic redox reactions.

## Graphical abstract

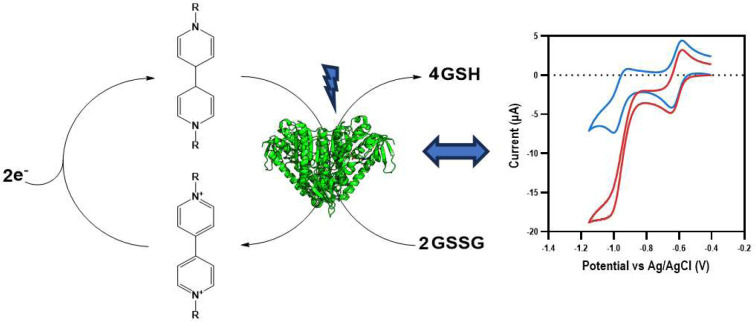

## Introduction

Due to the effect of greenhouse gas emissions and climate change on the environment, enabling a shift to more sustainable chemical production^1,2^ has been a major global research focus in recent years.^1^ Enzyme-based biocatalysis is becoming increasingly attractive for sustainable industrial processes, due to high selectivities and efficiencies coupled with powerful engineering techniques that allow for designing broad reactivities^3–5^ beyond the natural capability of the chosen enzyme. Despite this potential, industrial uptake of biocatalysis remains limited to the production of a relatively small proportion of high value chemicals.^6–9^ One of the major drawbacks for full deployment of enzymes is the frequently prohibitively expensive requirement for co-factors, and their associated regeneration.^10^ This is particularly true for enzymes that catalyse oxidation and reduction (redox) reactions, processes of critical importance to a vast range of chemical syntheses.^7,8^ Bioelectrocatalysis, or enzymatic catalysed electrochemical reactions at electrode surfaces,^11,12^ is a powerful approach to circumvent the requirement for cellular processes for co-factor regeneration. Oxidoreductases are ubiquitous enzymes that catalyse oxidation and reduction reactions that are coupled with cofactors such as NADH and NADPH. However, the expense and instability of redox cofactors traditionally used (*e.g.*, NADH and NADPH), coupled with the high over-potentials required to facilitate the hydride ion transfer and challenging regeneration,^10,13^ significantly hinders the development of new bioelectrocatalysis reactions. As such, there remains a great impetus to develop new mediators with both efficient electron transfer capabilities and facile electrochemical regeneration that can replace the need for NADPH.

Viologens, or 1,1′-disubstituted-4,4′-bipyridinium salts, are a class of organic redox active species that are promising alternative mediators to replace NADPH/NADH for redox enzymes.^14,15^ Viologens can exist in three distinct redox states; the dicationic oxidized state, the partially (1 electron) reduced radical cation state, and the fully (2 electron) reduced neutral state.^16^ The negative reduction potentials of viologens make them highly suited to mediate redox reactions taking place in flavin adenine dinucleotide (FAD)-containing enzymes, a common and important class of oxidoreductase enzyme. Furthermore, conventional viologens, such as methyl viologen, have been successfully used with a range of redox enzymes including lipoamide dehydrogenase,^17^ ferredoxin reductase,^17^ nitrogenase,^18^ [NiFe] hydrogenase,^19^ diaphorase,^20^ glucose oxidase,^21^ and metal-containing and metal-free formate dehydrogenases.^22,23^ Importantly, viologens are electrochemically and thermally stable, have highly modifiable chemical structures, and have a relatively cheap and facile synthesis.^16,24,25^ To date, most studies focus on the radical cation species of viologens as they are mostly soluble in aqueous systems, as compared to the fully reduced state which is typically water-insoluble.^23,26–28^ Whilst some research has been done on the electron mediating potential of fully reduced viologens,^14,29^ due to the insolubility of fully reduced viologens this work is limited. Altering the chemical moieties around the viologen core can greatly influence its solubility, particularly of the fully reduced species in an aqueous medium.^29^ However, this crucial aspect remains critically understudied. This untapped potential represents a new window of electrochemical opportunity for the use of viologens as NADH/NADPH replacements in bio-electrocatalysis.

**Fig. 1:**
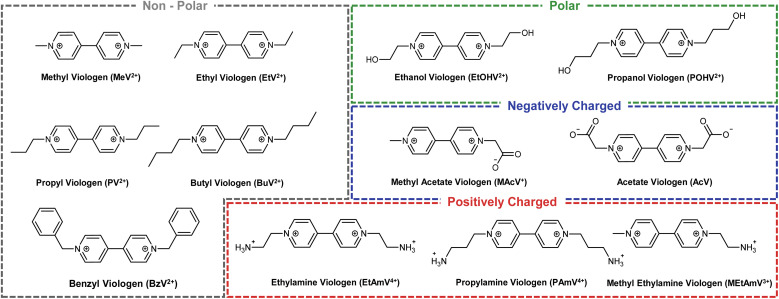
Viologen library. Chemical structures of the synthesized or commercially obtained viologens used in this study to examine effect of physicochemical properties on electrochemical mediation of enzymatic redox reactions.

Here, we present a rationally designed selection of viologens covering a range of polarities and functional groups (Fig. 1). The purpose of which is to fill the gap in knowledge between the physicochemical properties (structure, size, hydrophobicity, charge *etc.*) and the electrochemical properties, with particular attention paid to their ability to mediate bio-electrocatalysis. Using the FAD-containing enzyme glutathione reductase, a ubiquitous flavoenzyme that catalyses the reduction of the disulfide bond of oxidized glutathione (GSSG) to glutathione (GSH), as a model system, we fully characterise the electrochemical behaviour of the viologens as well as their potential to mediate bioelectrocatalysis. We show that through the modification of the viologen sidechain, it is possible to fine tune the first reduction potential (*E*_red,1_) of viologen to between −0.54 V and −0.67 V *vs.* Ag/AgCl. Subsequently, we then show that bioelectrocatalysis can be mediated at an increasing rate at more negative potentials, with electrostatic interactions representing an important secondary factor. In discovering that reduction potential was the defining characteristic of controlling the enzymatic reaction, we then investigated the second reduction potential (*E*_red,2_) of viologens for bio-electrocatalytic efficiency. Specifically, due to the enhanced solubility of a subset of viologens, values of *E*_red,2_ as low as −0.99 V *vs.* Ag/AgCl was possible, resulting in all viologens soluble in this redox state acting as highly effective mediators. These experiments therefore provide a blueprint for the re-alignment of the paradigm of using viologens for bioelectrocatalysis, paving the way for a cost-effective strategy for the full deployment of redox enzymes in sustainable chemical production, and moving viologen from very structure-dependent 1-electron mediators to largely structure-independent 2-electron mediators.

## Experimental

### Chemicals

Unless stated otherwise, all chemicals were used as received. 4-4′ Dipyridyl (98%, Sigma Aldrich), acetonitrile (ACN, HPLC quality 99%, Sigma Aldrich), *N*,*N*-dimethylformamide (DMF, >99%, Fischer Chemical), deuterium oxide (D_2_O, 99.9%, Sigma Aldrich), dimethyl sulfoxide-d6 (DMSO-d6, 99.9%, Sigma Aldrich), ethanol absolute (99.8%, Sigma Aldrich), potassium chloride (99.0%, Sigma Aldrich), ethyl viologen dibromide (EtV, 99%, Sigma Aldrich), methyl viologen dibromide (MeV, 99%, Sigma Aldrich), benzyl viologen dibromide (BzV, 99%, Sigma Aldrich), l-glutathione oxidised disodium salt (99.8%, Sigma Aldrich), NADPH tetrasodium salt (>95%, Santa Cruz Biotechnology), 1 bromobutane (99.0%, Sigma Aldrich), 1-chloropropane (99.0%, Sigma Aldrich), iodopropane (99.0%, Sigma Aldrich), 2-bromoethanol (99.0%, Sigma Aldrich), bromoacetic acid (99.0%, Sigma Aldrich), 6-bromohexanoic acid (99.0%, Sigma Aldrich), (2-bromoethyl)trimethylammonium bromide (99.0%, Sigma Aldrich), glutathione reductase (GR) from baker's yeast (*S. cerevisiae*) in 3.6 M ammonium sulphate suspension (300 units per mg protein, 6.5 mg mL^−1^, 60 kDa (monomer),^30^ Sigma Aldrich).

### Synthesis of viologens

Methyl, ethyl and benzyl viologens were commercially obtained while the remaining viologens were successfully synthesized by nucleophilic substitution reaction of 4,4′-bipyridine,^24,31,32^ with a range of alkylating agents and leaving groups to afford mono- and di-substituted species. The viologens were all synthesized employing a variety of solvents, temperatures and times (see ESI Table 1† for further details).They were recrystallised using acetonitrile. The ^1^H NMR spectra of the commercial and synthesized viologens, and 4,4′-bipyridine, were recorded on a 400 MHz NMR machine using DMSO-d6 or D_2_O as the solvent (ESI Table 2†).

### NADPH-inhibition study

The absorbance of NADPH was monitored at 340 nm using a UV-vis spectrophotometer (Shimadzu UV-2600i) during its reaction time with GR for GSSG reduction in the presence or absence of viologen. The rate of change in absorbance was then used to calculate the rate at which the enzymatic reaction consumed NADPH (*ε*_340_ = 6.22 mM^−1^ cm^−1^).^33^ The uninhibited control reaction contained 10 μM GR, 10 mM GSSG, and 0.2 mM NADPH in 1 ml of 0.2 M Tris/KCl buffer at 30 °C. The inhibited reactions contained 10 μM GR, 10 mM GSSG, 0.2 mM NADPH, and 1 mM viologen in 1 ml of 0.2 M Tris/KCl buffer at 30 °C. The NADPH inhibition study contained 10 μM of GR, 10 mM of GSSG, 1 mM viologen, and 0.025–0.3 mM NADPH in 1 ml of 0.2 M Tris/KCl buffer at 30 °C. The GSSG inhibition study contained 10 μM of GR, 0.125–1 mM GSSG, 1 mM viologen and 0.2 mM NADPH in 1 ml of 0.2 M Tris/KCl buffer at 30 °C.

### Cyclic voltammetry

Cyclic voltammetric measurements were carried out using NOVA software (Metrohm Autolab, the Netherlands) in a three-electrode configuration: a 3.0 mm diameter glassy carbon working electrode (BASi, USA), a platinum counter electrode, and an Ag/AgCl reference electrode (3.5 M KCl, BASi, USA). All tabulated, plotted and stated values are as recorded *vs.* Ag/AgCl, unless otherwise stated; in order to convert recorded potential values to values *vs.* SHE, please add +0.210 V to the potential.^34^ The electrodes were thoroughly cleaned before every scan with alumina and deionised water.

Redox activity of all the viologens were assessed using a solution (5 mL) of 0.2 M Tris/KCl buffer, as the supporting electrolyte and 1 mM of the viologen. To confirm that the observed bioelectrocatalysis was a direct result of the enzyme, CV controls were performed with GSSG only, viologens with GSSG, GR only, GSSG and GR and Tris/KCl (ESI Fig. 1†). In all cases, without the enzyme, no electrocatalytic behaviour was observed.

For the bio-electrocatalytic activity of GR, the experimental cell was made up of a 20 ml sealed glass vial containing 200 μL of 1 mM viologen, 10 mM GSSG and 0.65 mg mL^−1^ GR in 0.2 M Tris/KCl buffer at pH 8. The tips of the reference, working, and counter electrodes were submerged in the 200 μL solution. The standard cell had the same set up with the absence of GR. CV experiments were recorded at 0.01 V s^−1^ unless stated otherwise. The CV experiments were performed under Ar and in triplicates (three new samples) with 2 standard deviations used as the error bars. The 1D diffusion boundary between the electrode surface and the walls of the cell was *ca.* 100 μm, which is relatively small given the diffusion coefficients of the viologen species and the scan rate. However, no evidence of thin-layer voltammetry was observed during this study.

### Digital simulation

Experimental data was simulated using the DigiElch software (Gamry Instruments, USA) to determine the thermodynamic and kinetic parameters such as reduction potentials (*E*_red,1_ and *E*_red,2_), diffusion coefficients (*D*), and bimolecular rate constants (*k*). Simulations were performed by fixing the transfer coefficient, *α*, at 0.5 (indicating the oxidation and reduction of the viologen was equally facile) and allowing the kinetic parameters to float. Diffusion coefficients of the viologens were in the range of 3.21–7.7 × 10^−6^ cm^2^ s^−1^ and rate constants were in the range of 0.001–0.014 cm s^−1^, in line with expected values. The diffusion coefficient of the GSH was taken from literature as 3.0 × 10^−6^ cm^2^ s^−1^.^35^ The diffusion coefficient of GR was determined to be 1.04 × 10^−6^ cm^2^ s^−1^ (using the Stokes–Einstein equation and a hydrodynamic radius of 2.35 nm, ESI Fig. 7†). The equilibrium constants used for the enzyme coupled reactions in the simulation were calculated using the reduction potentials of the viologens (Table 1), GSSG (−0.299 V *vs.* SHE),^36^ and GR (−0.360 V *vs.* SHE).^37^ The simulations were done based on principles of kinetic systems and electrochemically mediated enzymatic reactions by Nicholson and Shain, and Antiochia *et al.*^38,39^ For enzyme coupled reactions, the following ping-pong mechanism was assumed:

1 M_ox_ + e^−^ → M_red_

2
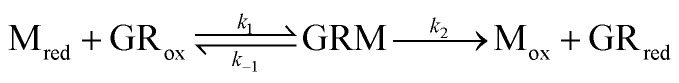
3
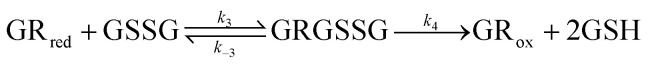
where M_ox_ and M_red_ are the oxidised and reduced mediator respectively; GR_ox_ and GR_red_ are the oxidised and reduced enzyme respectively; and GRM and GRGSSG are the enzyme-mediator and enzyme-substrate complexes respectively. Where *k*_1_, *k*_2_, *k*_3_ and *k*_4_ are the forward bimolecular rate constants while *k*_−1_ and *k*_−3_ are the backward bimolecular rate constants of their respective equations.

**Table 1:** Reduction potentials (*E*_red_) mediated current ratios (*I*_lim_/*I*_p_), diffusion coefficients (*D*), and corresponding bimolecular rate constants (*k*_1_) values for the viologens at their respective first and second reduction potentials^a^

Viologen	*E*_red,1_ (V)	*I*_lim,1_/*I*_p,1_	*D*_1_ × 10^−6^ (cm^2^ s^−1^)	*k*_1,1_ (M^−1^ s^−1^)	*E*_red,2_ (V)	*I*_lim,2_/*I*_p,2_	*D*_2_ × 10^−6^ (cm^2^ s^−1^)	*k*_1,2_ (M^−1^ s^−1^)
MAcV	−0.67	2.50 ± 0.13	7.42 ± 0.07	5300 ± 100	—	—	—	—
AcV	−0.67	2.37 ± 0.14	5.63 ± 0.67	4000 ± 300	—	—	—	—
MeV	−0.67	2.26 ± 0.07	6.63 ± 0.45	3800 ± 200	—	—	—	—
EtV	−0.67	1.54 ± 0.003	4.81 ± 0.11	1442 ± 2	—	—	—	—
PV	−0.66	1.17 ± 0.19	3.61 ± 0.08	780 ± 5	—	—	—	—
BuV	−0.66	1.13 ± 0.05	3.90 ± 0.01	298 ± 38	—	—	—	—
EtOHV	−0.61	1.05 ± 0.01	5.37 ± 0.35	196 ± 42	−0.96	2.56 ± 0.06	7.71 ± 0.01	10 000 ± 200
POHV	−0.63	1.04 ± 0.02	3.76 ± 0.01	126 ± 6	−0.96	2.23 ± 0.01	6.37 ± 0.01	5000 ± 700
MEtAmV	−0.60	1.12 ± 0.03	6.27 ± 0.01	293 ± 47	−0.91	1.91 ± 0.06	7.71 ± 0.01	3700 ± 0.5
EtAmV	−0.56	0.98 ± 0.02	4.94 ± 0.01	N/A^a^	−0.87	2.85 ± 0.06	5.33 ± 0.01	7000 ± 50
PAmV	−0.60	1.00 ± 0.01	3.30 ± 0.01	N/A^a^	−0.92	3.06 ± 0.09	3.37 ± 0.01	10 000 ± 50
BzV	−0.54	0.93 ± 0.03	3.54 ± 0.46	N/A^a^	—	—	—	—

a N/A – *k* values below 1 M^−1^ s^−1^ and therefore assumed negligible.

### NADPH-inhibition factor (NIF)

As NADPH was inhibited by the viologens at the GR active site, NIF was used as a parameter to determine the magnitude of binding of viologens at the active sites of the GR. NIF was calculated as the ratio of the rate of change in NADPH concentration with GR and GSSG in the absence of viologen to the rate of change in NADPH with GR and GSSG in the presence of viologen. This ratio resulted in an indicative value for inhibition, where NIF >1 indicates binding potential of the viologen at the active sites of the GR. NIF was the plotted against *I*_lim,1_/*I*_p,1_ (ESI Fig. 4†). Where *I*_lim,1_ is the maximum mediating current and *I*_p,1_ is the peak current of the same viologen in the absence of GR at the 1st redox process.

## Results and discussion

**Fig. 2:**
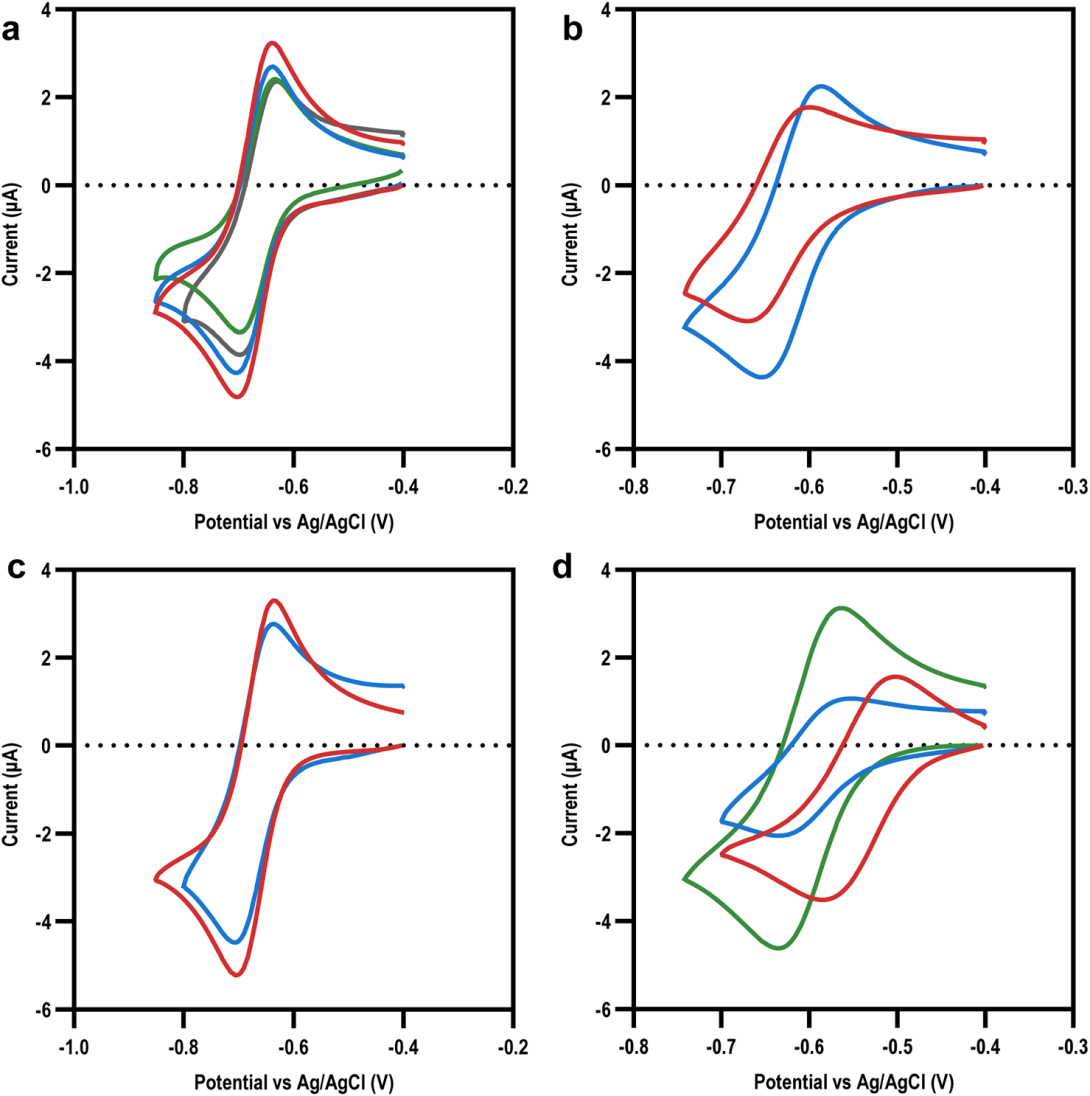
Electrochemistry of viologen library at first reduction potential. Cyclic voltammograms showing first redox peaks for: (a) non-polar viologens (MeV – red, EtV – blue, PV – green, BuV – grey); (b) polar viologens (EtOHV – red, and POHV – blue); (c) negatively charged viologens (AcV – blue, and MAcV – red); and (d) positively charged viologens (EtAmV – red, PAmV – blue, and MEtAmV – green).

Once our library of viologens (Fig. 1) had been successfully synthesized (ESI Table 1†) and confirmed as pure (ESI Table 2†), cyclic voltammetry (CV) was used to examine their electrochemical behaviour (Fig. 2). Simulation of the experimental data was used to extract diffusion coefficients, bimolecular rate constants (*k*_1_), and reduction potentials (ESI Fig. 1†). The radicals (first reduction state) of all the viologens were soluble at the electrode–electrolyte interface with the exception of BzV, which due to the hydrophobic nature of the benzyl rings precipitated out of solution once reduced from the dication to the radical (ESI Fig. 1†). The library of viologens exhibited a broad range of potentials, with the first reduction potential (*E*_red,1_) ranging from a minimum of −0.54 V *vs.* Ag/AgCl for BzV to a maximum of −0.67 V for MeV, EtV, MAcV and AcV. These potentials were consistent with previous reports,^16,23,40^ with the breadth of potentials clearly demonstrating the fine-tuning capability available within our library. Given that the *E*_red,1_ of the viologens in our library were on par with, or more negative than, the redox potential of NADPH (−0.52 V to −0.57 V *vs.* Ag/AgCl)^37^ we decided they were all potentially good candidates to mediate electron transfer to enable bio-electrocatalysis.

**Fig. 3:**
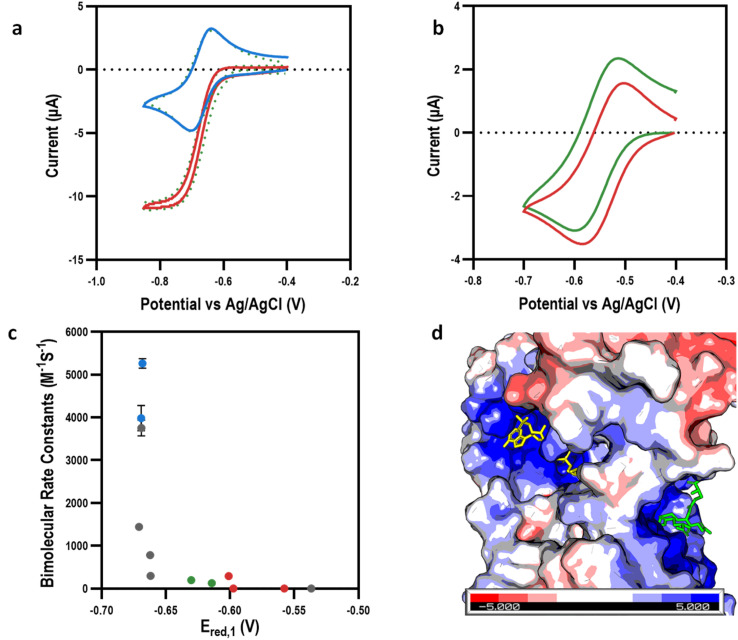
Mediating bio-electrocatalysis at the first reduction potential. (a) Cyclic voltammograms showing example of bio-electrocatalytic reduction of GSSG by GR with MeV as the electron transfer mediator: without GR (blue); with GR (red); simulation (dotted green lines). (b) Cyclic voltammograms showing example of no bio-electrocatalytic reduction of GSSG by GR was observed with EtAmV as the electron transfer mediator (green) with control reaction for reference (red). (c) Bimolecular rate constant (*k*_1_) (at *E*_red,1_) for the viologen library: MAcV and AcV (blue); alkyl and benzyl viologens (grey); EtOHV and POHV (green); EtAmV, PAmV, and MetAmV (red). (d) Surface representation of GR (PDB: 2HQM^41^) showing positive charges surrounding GR active site with GSSG (green) and NADPH (yellow) shown at their respective binding sites.

To investigate how the *E*_red,1_ and other physicochemical properties of our viologens might affect bioelectrocatalysis mediation, we turned to the well characterised reduction of di-glutathione (GSSG) by the enzyme glutathione reductase (GR) as a model system for study (Fig. 3a). CV experiments were performed on the library of viologens in the presence of GSSG; no electrocatalysis was observed in the absence of GR indicating the enzyme was necessary, with no direct viologen-GSSG electro catalysis occurring (ESI Fig. 3†). Viologen-mediated bio-electrocatalytic reduction of GSSG by GR was observed by cyclic voltammetry in the presence of all the viologens apart from BzV and the tetra-cationic viologens, EtAmV and PAmV (Fig. 3a and b, ESI 3†).

To account for differences in physicochemical properties, such as diffusion, the observed mediating capability of the viologens was initially quantified using a ratio of the maximum mediating current, *I*_lim_, to the peak current of the same viologen in the absence of GR, *I*_p_ (Table 1). This ratio is well established to be proportional to the rate of the mediation process.^38,39^ Having established the mediation capabilities of our viologens, we then used data simulation to estimate the bimolecular rate constant (*k*_1_) of the enzyme-coupled reactions (ESI Fig. 8 and 9†). As expected, a strong correlation was observed between *I*_lim_/*I*_p_ and *k*_1_. Plotting *k*_1_ against *E*_red,1_ (Fig. 3c) revealed a very strong initial dependence on the reduction potential for mediating the biocatalytic reduction of GSSG, with only *E*_red,1_ reduction potentials more negative than −0.6 V effectively mediating the reaction. The *k*_1_ values were in the range of 0.1–5300 M^−1^ s^−1^, which was consistent with previous literature values of MeV with the enzyme diaphorase.^20^ It was also apparent that *k*_1_ was influenced to a degree by structural factors. For example, MeV and EtV were able to mediate bioelectrocatalysis to a high degree, with *k*_1_ values of 3754 ± 188 and 1442 ± 2 M^−1^ s^−1^ respectively, whereas the larger PV and BuV were significantly less effective despite similar *E*_red,1_ values, indicating a steric effect.

The highest *k*_1_ values were calculated for the zwitterionic viologens MAcV and AcV at 5300 ± 100 and 4000 ± 300 M^−1^ s^−1^ respectively. Interestingly, the smaller positively charged viologens (*e.g.*, MeV^2+^) or more highly charged viologens (*e.g.*, EtAmV^4+^) did not mediate the reaction as effectively. This suggested that charge attraction/repulsion at the active site was playing a role. Inspection of the crystal structure of GR revealed that this was indeed the case; the highly positively charged nature of the GR binding pocket (comprising of both GSSG and NADPH active sites) was attributed to both the strong mediation by negatively charged viologens and the no observable mediation by positively charged viologens (Fig. 3d). Intriguingly, despite their hydroxyl moieties, the polar viologens (EtOHV and POHV) exhibited poor electron mediating capabilities with respective *k*_1_ values of 196 ± 42 and 126 ± 6 M^−1^ s^−1^. The value of *E*_red,1_ was therefore an initial determining factor if the viologen had a sufficient over-potential to mediate the enzymatic reaction, with a secondary dependence on the overall charge of the viologens and tertiary dependence on the size of the viologen (Table 1).

To investigate any further explanations for the observed mediating capabilities, NADPH and GSSG inhibition studies were conducted (ESI Fig. 4†). UV-vis kinetic experiments revealed that GSSG binding to GR was competitively inhibited by the viologens (ESI Fig. 4a†), whilst NADPH binding to GR was largely uncompetitively inhibited (ESI Fig. 4b†). This suggested a potential shift in mechanism for electrochemically mediated reactions when compared to NADPH mediated reactions, which warrants further study. Nevertheless, it was observed that the various viologens inhibited GSSG and NADPH to varying degrees, however, plotting *I*_lim_/*I*_p_ or *k*_1_ against NADPH inhibition showed no discernible trend (ESI Fig. 4c†). This confirmed that the viologens were indeed binding to the active site of GR, but that the strength of that interaction was not a determining factor in the efficiency of electron transfer from the reduced viologens to the GR active site. This further indicated that although the chemical properties of viologens played a role, the reduction potential was seen to be the primary determining factor for electron mediation by the viologen, with electrostatic and steric properties as secondary factors.

**Fig. 4:**
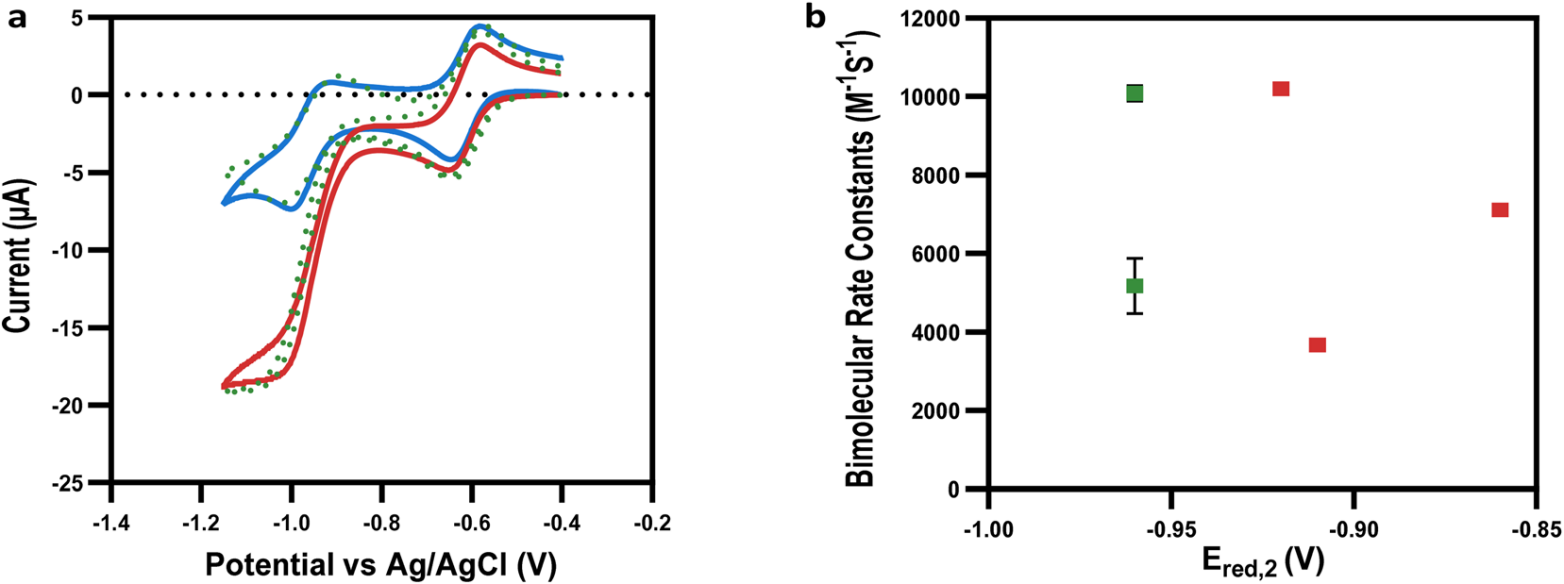
Mediating bio-electrocatalysis at the second reduction potential. (a) Cyclic voltammogram showing example of bio-electrocatalysis at the second reduction potential of EtOHV with GSSG in the presence of (red) or absence (blue) of GR (simulation – green dotted lines). (b) Comparison of bimolecular rate constants (*k*_1_) at *E*_red,2_: MAcV and AcV (blue); alkyl and benzyl viologens (grey); EtOHV and POHV (green); EtAmV, PAmV and MetAmV (red).

Given the strong dependence of *E*_red,1_ on mediation, we hypothesized that using viologens at the second reduction potential (*E*_red,2_) could further increase mediation capability. CV experiments of the viologens were performed to determine their solubility and electrochemical properties at lower reduction potentials, *E*_red,2_ (−0.74 V to −0.99 V *vs.* Ag/AgCl), with diffusion coefficients, and bimolecular rate constants extracted by simulating the experimental data (ESI Fig. 5 and 8†). In line with previous results,^16^ when reduced to the fully reduced (neutral) state, the non-polar and negatively charged viologens were insoluble or sparingly soluble, making them unsuitable for mediating bio-electrocatalysis. However, in contrast, the polar (EtOHV and POHV) and positively charged (MEtAmV, EtAmV and PAmV) viologens remained soluble in their fully reduced states. We therefore performed CV experiments on these fully reduced but soluble viologens (*E*_red,2_ from −0.92 V to −0.96 V *vs.* Ag/AgCl) in the presence of GSSG and GR to investigate their utility for mediating bioelectrocatalysis (Fig. 4a and ESI 6†).

These experiments, that to the best of our knowledge are the first of their kind, demonstrated that all of the viologens that were soluble in the fully reduced state were also capable of mediating the biocatalytic reduction of GSSG by GR (Fig. 4a). Interestingly, every viologen soluble in its di-reduced state acted as an effective mediator. Unlike what was observed for bioelectrocatalysis at *E*_red,1_, there was no correlation between the *E*_red,2_ and either *I*_lim,2_/*I*_p,2_ or *k*_1_ (Fig. 4b), indicating decreased structure sensitivity. This is likely the result of achieving a sufficient over-potential with respect to this reaction. Specifically, high *k*_1_ values were determined at *E*_red,2_ for the positively charged (10 000 ± 50, 7000 ± 5, and 3700 ± 0.5 M^−1^ s^−1^ for PAmV, EtAmV and MetAmV respectively) and polar viologens (10 000 ± 191 and 5000 ± 700 M^−1^ s^−1^ for EtOHV and POHV respectively). This was in contrast to their low *k*_1_ values (196 ± 42, 126 ± 6, 0.1, 0.1 and 293 ± 47 M^−1^ s^−1^ for EtOHV, POHV, PAmV, EtAmV and MEtAmV respectively) when mediating the reaction *via E*_red,1_. As such, we demonstrated that viologens with enhanced solubility at the fully reduced state were able to mediate bioelectrocatalysis effectively and seemingly, independent of other chemical properties. This was particularly evident for the positively charged viologens, which were now the most effective, strongly indicative of a shift to a more structurally independent electron transfer. The main potential caveat for using fully reduced viologens to mediate this reaction is regarding the increased overpotential required for effective biocatalysis. When compared to the potential for the GR enzyme (*ca.* −0.57 V *vs.* Ag/AgCl), the required *E*_red,1_ for appreciable bioelectrocatalysis using viologens was −0.67 V, which represented a *ca.* 0.1 V overpotential. Moving to the range of *E*_red,2_ values that demonstrated bioelectrocatalysis (*ca.* −0.91 V to −0.97 V), these are effectively an overpotential of 0.24–0.30 V. However, the highest bimolecular rate constant at *E*_red,2_ (10 000 M^−1^ s^−1^) was also 2-fold higher than the highest at *E*_red,1_ (5300 M^−1^ s^−1^). Furthermore, in the case of EtOHV, an overpotential of 0.39 V saw a 51-fold increase in bimolecular rate constant (from 192 M^−1^ s^−1^ to 10 000 M^−1^ s^−1^) at the two-electron reduction state of the viologen. As such, stabilizing the second redox state of viologens can be seen as a powerful design parameter for efficiently mediating bioelectrocatalysis.

## Conclusion

In conclusion, through the rational design and creation of a diverse library of viologens, we have demonstrated that reduction potential is the defining characteristic for mediating bio-electrocatalytic reactions with high efficiencies. Specifically, using the reduction of GSSG by GR as a model reaction, we showed that electron transfer to the FAD at the active site of GR was highly sensitive to the reduction potential of the viologen. Additionally, the kinetics of the reaction could be finely tuned through the chemical properties of the modified viologens, and particularly how they interact with the surface topology of the enzyme. Critically, by modifying viologen so that it remained soluble in the fully reduced form, we were able to perform highly efficient bioelectrocatalysis using the second redox potential of viologen; the first time fully reduced viologens have been reported to mediate electron transfer to a flavoenzyme. As such, through demonstrating an unprecedented level of control over the available window of reduction potentials, alongside a widening of the useable range of overpotentials, we have provided a blueprint for reimagining the use of viologens in bio-electrocatalysis. In particular, this work demonstrates the potential of viologen mediators for replacing NADPH (and associated recycling) in promoting biocatalytic redox reactions. The simplicity of this reaction system in circumventing the requirement for expensive co-factors, will be of great benefit to those working on industrially relevant transformations with a wide range of redox enzymes. Investigating the mechanism of the electron transfer process at the second reduction potential and deciphering whether it is truly independent of the structural binding of the viologen at the active site of flavoenzyme, will be the focus of future work.

## Data availability

All data are available in the main text or the ESI,† and are otherwise available upon reasonable request from the corresponding author.

## Author contributions

All authors contributed to the design and analysis of the experiments. D. A. K. synthesized and characterized all viologens, performed and analysed all electrochemical experiments, and wrote the manuscript. J. H. N. performed and analysed inhibition experiments and edited the manuscript. A. P. S. B. and L. A. conceived the idea and edited the manuscript.

## Conflicts of interest

The authors declare no competing interests.

## Supplementary Material

SC-015-D4SC02431A-s001

## References

[cit1] Hoekstra A. Y., Mekonnen M. M. (2012). Proc. Natl. Acad. Sci. U. S. A..

[cit2] Sheldon R. A. (2017). Green Chem..

[cit3] Brogan A. P. S., Bui-Le L., Hallett J. P. (2018). Nat. Chem..

[cit4] Arnold F. H. (2018). Angew. Chem., Int. Ed..

[cit5] Sheldon R. A., Woodley J. M. (2018). Chem. Rev..

[cit6] Pellis A., Cantone S., Ebert C., Gardossi L. (2018). New Biotechnol..

[cit7] Adams J. P., Brown M. J. B., Diaz-Rodriguez A., Lloyd R. C., Roiban G. D. (2019). Adv. Synth. Catal..

[cit8] Wu S., Snajdrova R., Moore J. C., Baldenius K., Bornscheuer U. T. (2021). Angew. Chem., Int. Ed..

[cit9] Yi D., Bayer T., Badenhorst C. P. S., Wu S., Doerr M., Höhne M., Bornscheuer U. T. (2021). Chem. Soc. Rev..

[cit10] Schulz M., Bayer M., White H., Günther H., Simon H. (1994). Biocatal. Biotransform..

[cit11] Guang L., Koomson D. A., Jingyu H., Ewusi-Mensah D., Miwornunyuie N. (2020). Int. J. Environ. Res. Public Health.

[cit12] Koomson D. A., Huang J., Li G., Miwornunyuie N., Darkwah W. K. (2022). Renewable Energy.

[cit13] Spaans S. K., Weusthuis R. A., van der Oost J., Kengen S. W. M. (2015). Front. Microbiol..

[cit14] Willner I., Riklin A., Kasher R., Katz E. (1992). J. Am. Chem. Soc..

[cit15] Gómez-Moreno C., Bes M. T. (1994). Biochim. Biophys. Acta, Bioenerg..

[cit16] Bird C. L., Kuhn A. T. (1981). Chem. Soc. Rev..

[cit17] DiCosimo R., Wong C. H., Daniels L., Whitesides G. M. (1981). J. Org. Chem..

[cit18] Milton R. D., Cai R., Abdellaoui S., Leech D., De Lacey A. L., Pita M., Minteer S. D. (2017). Angew. Chem., Int. Ed..

[cit19] Lielpetere A., Becker J. M., Szczesny J., Conzuelo F., Ruff A., Birrell J., Lubitz W., Schuhmann W. (2022). Electrochem. Sci. Adv..

[cit20] Kim S., Yun S. E., Kang C. (1999). J. Electroanal. Chem..

[cit21] Liu X., Neoh K. G., Cen L., Kang E. T. (2004). Biosens. Bioelectron..

[cit22] Jayathilake B. S., Bhattacharya S., Vaidehi N., Narayanan S. R. (2019). Acc. Chem. Res..

[cit23] Miyaji A., Amao Y. (2020). New J. Chem..

[cit24] Komoda K., Kawauchi T. (2021). Polym. J..

[cit25] Liu M. O., Chen I. M., Lin J. L. (2007). Mater. Lett..

[cit26] Ding J., Zheng C., Wang L., Lu C., Zhang B., Chen Y., Li M., Zhai G., Zhuang X. (2019). J. Mater. Chem. A.

[cit27] Badalyan A., Yang Z. Y., Hu B., Luo J., Hu M., Liu T. L., Seefeldt L. C. (2019). Biochemistry.

[cit28] Wang X., Guo L., Cao S., Zhao W. (2020). Chem. Phys. Lett..

[cit29] Schmedding D. J. M., Somers W., van Dijk C., Günther H., van der Lugt J. P., Simon H. (1992). Biotechnol. Tech..

[cit30] Mavis R. D., Stellwagen E. (1968). J. Biol. Chem..

[cit31] Park K. K., Han S. Y., Park Y. H., Park J. W. (1997). Tetrahedron Lett..

[cit32] Striepe L., Baumgartner T. (2017). Chem.–Eur. J..

[cit33] Veskoukis A. S., Margaritelis N. V., Kyparos A., Paschalis V., Nikolaidis M. G. (2018). Redox Rep..

[cit34] Friis E. P., Andersen J. E. T., Madsen L. L., Bonander N., Moller P., Ulstrup J. (1998). Electrochim. Acta.

[cit35] Oyesanya O. , Mechanistic Studies on the Electrochemistry of Glutathione and Homocysteine, 2008

[cit36] Khazim K., Giustarini D., Rossi R., Verkaik D., Cornell J. E., Cunningham S. E. D., Mohammad M., Trochta K., Lorenzo C., Folli F., Bansal S., Fanti P. (2013). Transl. Res..

[cit37] Veine D. M., Arscott L. D., Williams C. H. (1998). Biochemistry.

[cit38] Nicholson R. S., Shain I. (1964). Anal. Chem..

[cit39] Antiochia R., Lavagnini I., Magno F. (2001). Electroanalysis.

[cit40] Dam v. H. T., Ponjeé J. J. (1974). J. Electrochem. Soc..

[cit41] Yu J., Zhou C. Z. (2007). Proteins: Struct., Funct., Genet..

