# Supplementary material for: Re-assessing viologens for modern bio-electrocatalysis
Source: Chem Sci. 2024 May 16;15(24):9325–32. doi: 10.1039/d4sc02431a (PMC11186337; doi:10.1039/d4sc02431a)
Supplement: SC-015-D4SC02431A-s001 [file SC-015-D4SC02431A-s001.pdf]

Electronic Supplementary Material (ESI) for Chemical Science.
This journal is © The Royal Society of Chemistry 2024

SUPPLEMENTARY INFORMATION

Re-assessing viologens for modern bio-electrocatalysis

Desmond Ato Koomson[1], Jake H. Nicholson[1], Alex P. S. Brogan[1,*], and Leigh Aldous[1,*]

1. Department of Chemistry, Britannia House, King's College London, London SE1 1DB, UK

Supplementary Table 1. Materials and conditions for synthesis of viologen library.

Viologens	Reagents	Mass/ Volume	Solvents	Temperature (ºC)	Duration	Observation
1-MBP	4,4'-bipyridine	5 g	Ethyl Acetate (50 mL)	4	1 Month	Orange precipitate
	Iodomethane	1.196 mL				
PV1	4,4'-bipyridine	1 g	ACN (10 mL)	60	24 hours	Colourless solution
	1-Chloropropane	1.691 mL	DMF (50 mL)	120	120 hours	Crystal yellow solution
PV 2	4,4'-bipyridine	1 g				
	Iodopropane	1.873 mL	ACN (50 mL)	70	210 hours	Red precipitates
BuV	4,4'-bipyridine	1 g	ACN (50 mL)	60	336 hours	Yellow precipitates
	1-Bromobutane	2.763 mL				
AcV	4,4'-bipyridine	1 g	ACN (75 mL)	90	22 hours	Crystal brown solution with brown precipitates
	Bromoacetic acid	2.669 g				
MAcV	Iodoacetic acid	0.8799 g	ACN (65 mL)	90	145 hours	Deep orange precipitates
	1-MBP	1.4106 g				
EtOHV	4,4'-bipyridine	1 g	ACN (50 mL)	60	120 hours	White precipitates
	2-Bromoethanol	1.588 mL	DMF (65 mL)	120	96 hours	Cream precipitates
POHV	4,4'-bipyridine	1 g	DMF (95 mL)	120	410 hours	Brown precipitates
	3-Bromo-1-propanol	2026.53 mL				
EtAmV	4,4'-bipyridine	1 g	DMF (70 mL)	120	168 hours	Brown precipitate formation
	2-Bromoethylamine hydrobromide	3.936 g				
PAmV	4,4'-bipyridine	1 g	DMF (95 mL)	120	550 hours	Light brown precipitate formation
	2-Bromopropylamine hydrobromide	4.205 g				

MEtAmV	1-MBP	1.1393 g	ACN (50 mL)	90	117 hours	Orange precipitate
	2-Bromoethylamine hydrobromide	1.566 g				

Supplementary Table 2. ^{1}H NMR (400 MHz) characterisation of viologen library.

Viologens	Proton (s)	Multiplicity	Chemical shift δ (ppm)	Solvent
Methyl Viologen (MeV)	2H	d	9.0	D_2O
	2H	d	8.46	
	3H	s	4.44	
Ethyl Viologen (EtV)	2H	d	9.37	DMSO-d6
	2H	d	8.76	
	2H	q	4.73	
	3H	t	1.67	
Propyl Viologen (PV)	2H	d	9.51	DMSO-d6
	2H	d	8.90	
	2H	t	4.74	
	2H	sex	2.03	
	3H	t	0.97	
Butyl Viologen (BuV)	2H	d	9.52	DMSO-d6
	2H	d	8.88	
	2H	t	4.77	
	2H	quin	1.97	
	2H	q	1.32	
	3H	t	0.91	
Benzyl Viologen (BzV)	2H	d	9.11	D_2O
	2H	d	8.48	
	2H	m	7.52	
	3H	s	5.84	
Ethanol Viologen (EtOHV)	2H	d	9.08	D_2O
	2H	d	8.51	
	2H	t	4.82	
	2H	t	4.11	
Propanol Viologen (POHV)	2H	d	9.10	D_2O
	2H	d	8.50	
	2H	t	4.80	
	2H	t	3.66	
	1H	s	2.66	
	2H	quin	2.27	
Acetate Viologen (AcV)	2H	d	9.02	D_2O
	2H	d	8.49	

	2H	s	4.48	
Methyl Acetate Viologen (MAcV)	2H	d	9.33	D_2O
	2H	d	8.82	
	2H	s	3.39	
	3H	s	2.52	
Ethyl Amine Viologen (EtAmV)	2H	d	9.20	D_2O
	2H	d	8.60	
	2H	t	5.06	
	2H	t	3.73	
	2H	s	1.97	
Propyl Amine Viologen (PAmV)	2H	d	9.13	D_2O
	2H	d	8.56	
	2H	t	4.81	
	2H	t	3.15	
	3H	s	2.65	
	2H	quin	2.45	
Methyl Amine Viologen (MEtAmV)	2H	d	9.18	D_2O
	2H	d	9.01	
	2H	d	8.61	
	2H	d	8.48	
	2H	t	5.07	
	3H	s	4.46	
	2H	t	3.75	

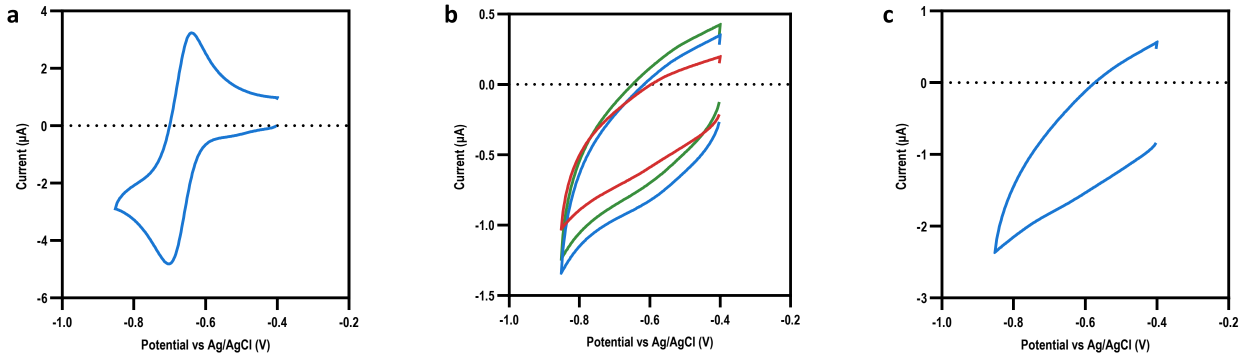

Supplementary Figure 1. Cyclic Voltammograms showing no bio-electrocatalytic activity: a – MeV and GSSG; b – GSSG only (red), GR only (green), and GSSG and GR (blue); c – Tris/KCl only.

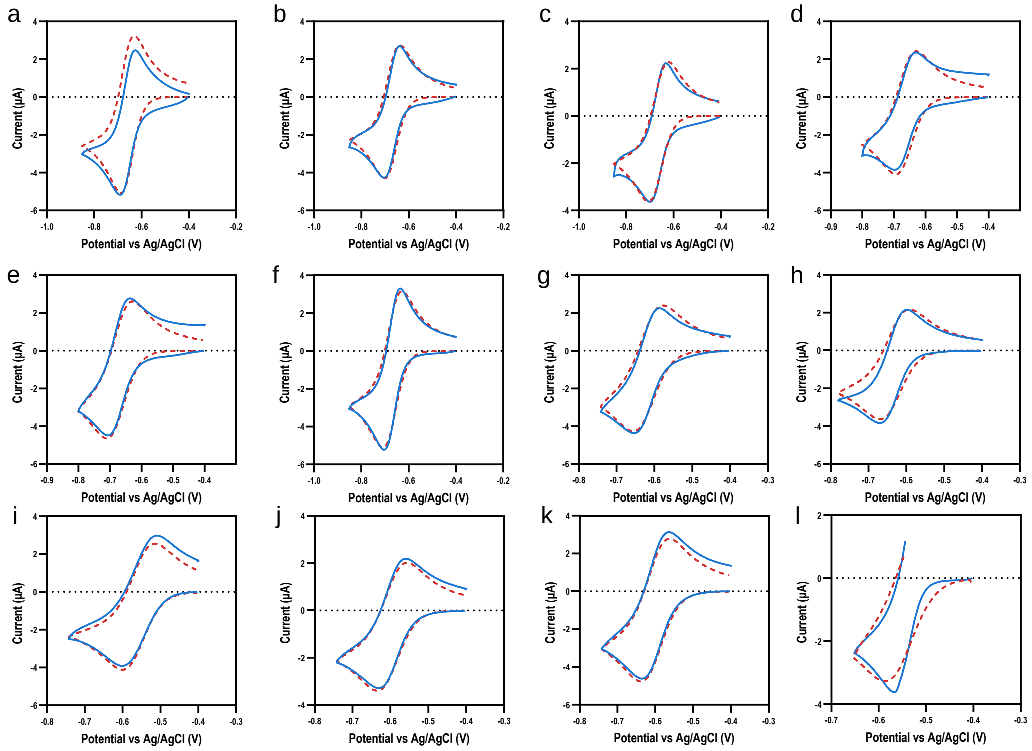

Supplementary Figure 2. First redox cyclic voltammograms (solid blue line) of viologens with their corresponding simulated fits (dotted red line): a – MeV; b – EtV; c – PV; d – BuV; e – AcV; f – MacV; g – EtOHV; h – POHV; I – EtAmV; j – PamV; k – MetAmV; and l – BzV.

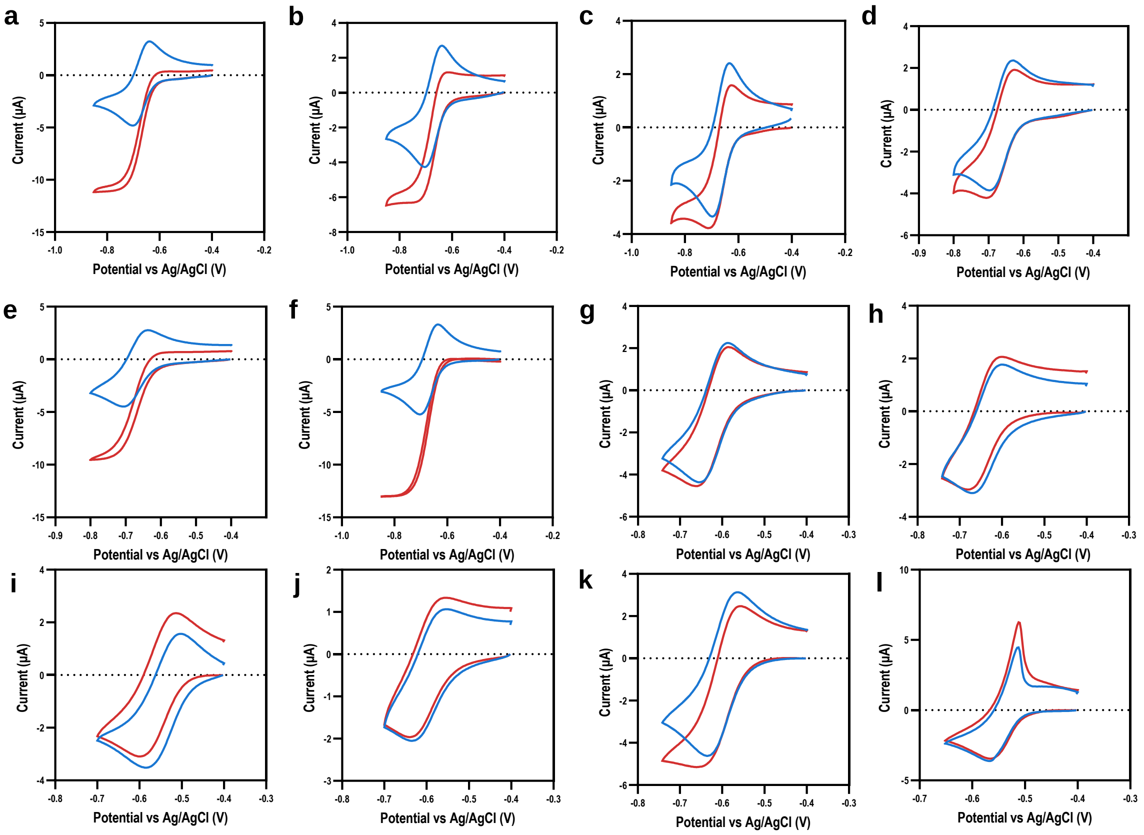

Supplementary Figure 3. Cyclic voltammograms showing viologens at $E_{red,1}$ with GSSG in the presence (red) or absence (blue) of GR for: a – MeV; b – EtV; c – PV; d – BuV; e – AcV; f – MacV; g – EtOHV; h – POHV; I – EtAmV; j – PamV; k – MetAmV; and l – BzV

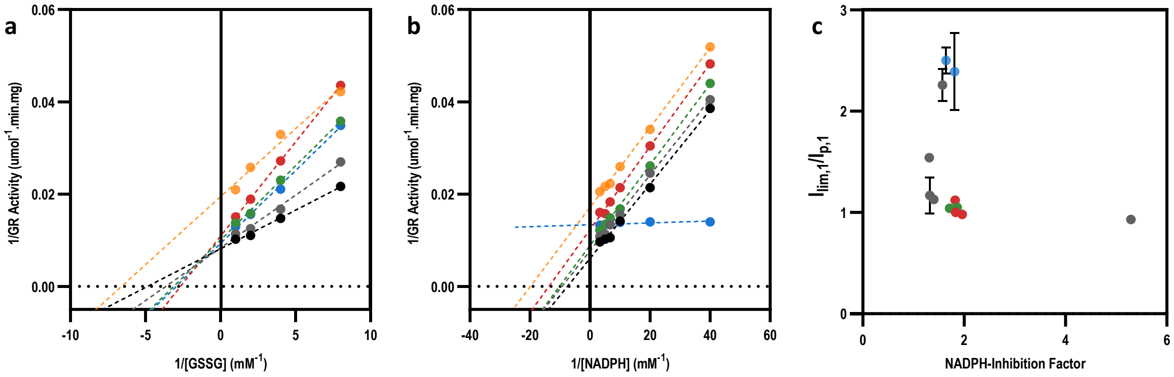

Supplementary Figure 4. a – Lineweaver-Burk plot showing inhibition of GSSG at GR active site in presence of viologens: uninhibited control (black), MeV (grey), BzV (orange), EtOHV (green), EtAmV (red), and AcV (blue); b – Lineweaver-Burk plot showing inhibition of NADPH at GR active site in presence of viologens: uninhibited control (black), MeV (grey), BzV (orange), EtOHV (green), EtAmV (red), and AcV (blue); c – $I_{lim,1}/I_{p,1}$ versus NADPH-inhibition factor of the viologens.

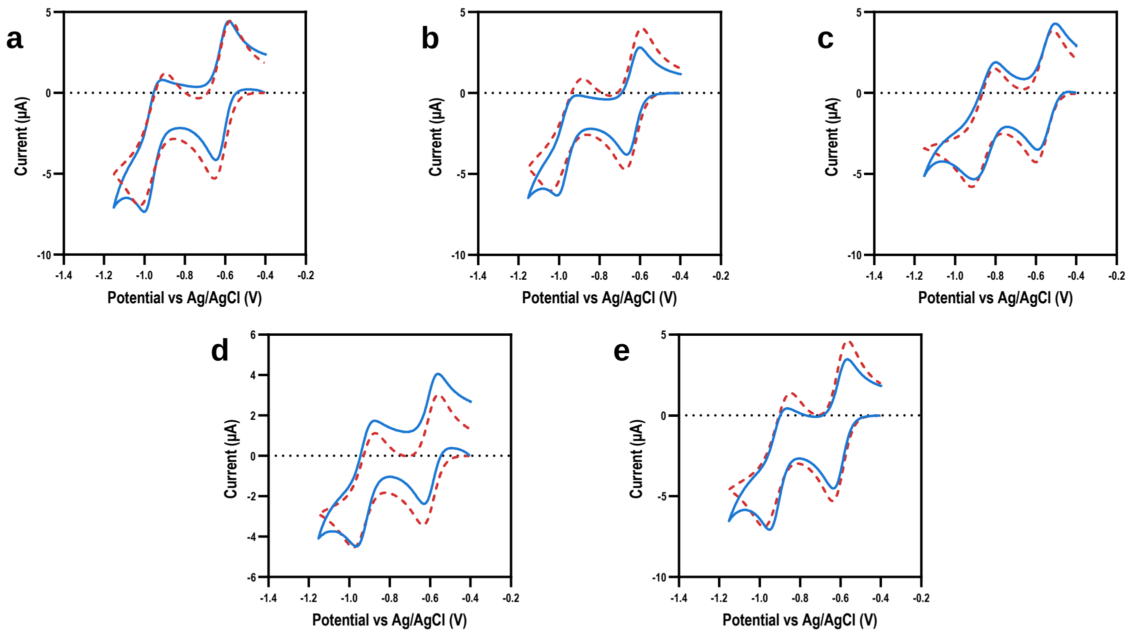

Supplementary Figure 5. Second redox cyclic voltammograms (solid blue line) of viologens with their corresponding simulated fits (dotted red line): a – EtOHV; b – POHV; c – EtAmV; d – PamV; e – MetAmV.

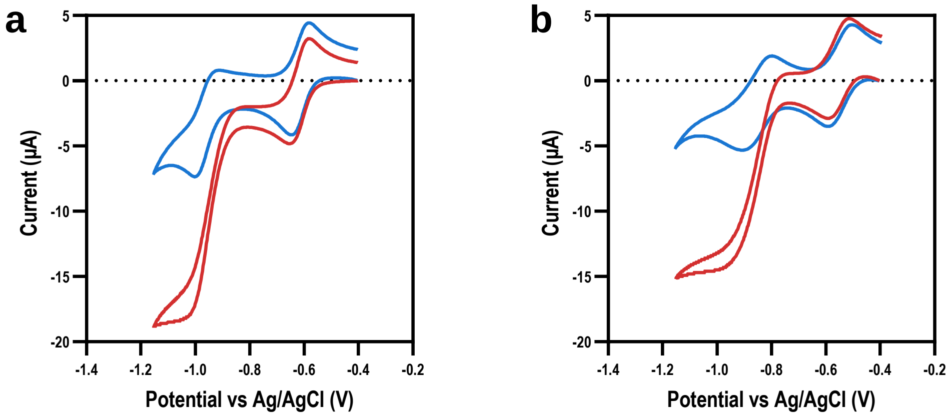

Supplementary Figure 6. Cyclic voltammograms showing polar and positively charged viologens at $E_{red,2}$ with GSSG in the presence (red) or absence (blue) of GR: a – Polar viologen (EtOHV); and b – Positively charged viologen (EtAmV).

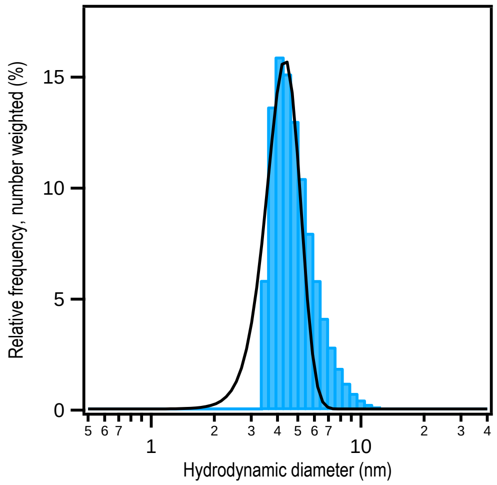

Supplementary Figure 7. Plot of number weight particle size distribution against hydrodynamic diameter, as collected by dynamic light scattering, for glutathione reductase in water (data fitted with Gaussian distribution, black, to determine average diameter).

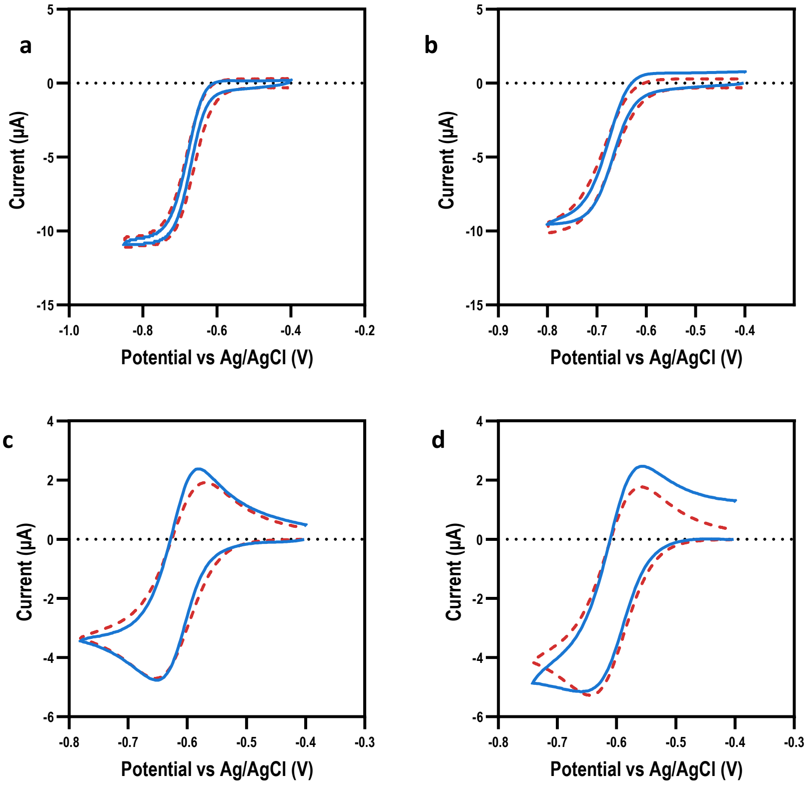

Supplementary Figure 8. First redox cyclic voltammograms (solid blue line) showing bio-electrocatalysis with their corresponding simulated fits (dotted red line): a – MeV; b – AcV; c – EtOHV; and d – MetAmV.

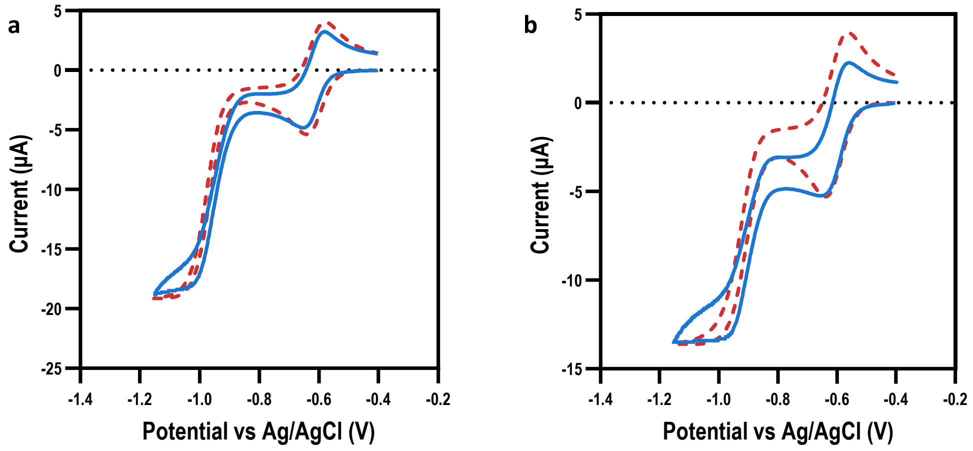

Supplementary Figure 9. Second redox cyclic voltammograms (solid blue line) showing bio-electrocatalysis with their corresponding simulated fits (dotted red line): a – EtOHV; and c – MetAmV.
